# A preliminary checklist of soil ants (Hymenoptera: Formicidae) of Colombian Amazon

**DOI:** 10.3897/BDJ.6.e29278

**Published:** 2018-11-07

**Authors:** Daniel Castro, Fernando Fernández, Andrés D Meneses, Maria C Tocora, Stepfania Sanchez, Clara P Peña-Venegas

**Affiliations:** 1 Instituto Amazónico de Investigaciones Científicas SINCHI, Leticia, Colombia Instituto Amazónico de Investigaciones Científicas SINCHI Leticia Colombia; 2 Instituto de Ciencias Naturales, Universidad Nacional de Colombia, Bogotá, Colombia Instituto de Ciencias Naturales, Universidad Nacional de Colombia Bogotá Colombia

**Keywords:** TSBF, Amazon basin, soil macrofauna, biogeography, species distribution.

## Abstract

**Background:**

This paper presents an updated list of soil ants of the Colombian Amazon collected in three different river basins: the Amazon, the Caquetá and the Putumayo. The list includes 10 subfamilies, 60 genera and 218 species collected from TSBF monoliths at four different depths (Litter, 0 - 10 cm, 10 - 20 cm and 20 - 30 cm). This updated list increases considerably the knowledge of edaphic macrofauna of the region, due to the limited published information about soil ant diversity in the Colombian Amazon region.

**New information:**

This is the first checklist of soil ant diversity of the Colombian Amazon region. Six new records of species for Colombia are exposed: *Acropyga tricuspis* (LaPolla, 2004), *Typhlomyrmex clavicornis* (Emery, 1906), *Typhlomyrmex meire* (Lacau, Villemant & Delabie, 2004), *Cyphomyrmex bicornis* (Forel, 1895), *Megalomyrmex emeryi* (Forel, 1904) and *Myrmicocrypta spinosa* (Weber, 1937), most of them corresponding to subterranean ants.

## Introduction

In tropical forests, the abundance and diversity of ants is usually high, which brings out the importance of ants for these ecosystems (Floren and Linsenmair 2005, Floren et al. 2002, Dunn et al. 2007, Davidson et al. 2007, Jaffe et al. 2007). Ants, together with earthworms and termites, are known as "ecosystem engineers" due to the positive effect of their activity on ecosystems (Decaëns et al. 1999, Decaëns 2010, Lavelle et al. 1997, Luke et al. 2014, Griffiths et al. 2017). Physical, chemical and biological soil properties are positively affected by the presence of ant nests, chambers, galleries and mineral aggregates that ants create (Seybold et al. 1999, Barros et al. 2001, Sanabria et al. 2014, Wu et al. 2015)

Diversity of soil ants in Amazonian forests is notoriously high (Ryder Wilkie et al. 2010, Ryder Wilkie et al. 2007, Bastos and Harada 2011, Bruna et al. 2008). In Colombia, ant lists include reports from coastal, mountain and Amazonian ecosystems (Pérez et al. 2009, Sanabria-Blandón and Achury 2011, Sanabria-Blandón and de Ulloa 2011, Vergara-Navarro and Serna 2013, Valdés-Rodríguez et al. 2014). However, information on soil ant diversity in the Colombian Amazon region is limited, due to the small number of works on this topic that have been published (Ospina and Fagua 2007).

This paper reports a preliminary checklist of soil ants collected in the Colombian Amazon region, with the purpose of contributing to a better understanding of the biogeographical distribution of these insects in the three most important river basins of the Amazon region of Colombia: the Amazon, the Caquetá and the Putumayo.

## Materials and methods

### Study area

**Figure 1.**
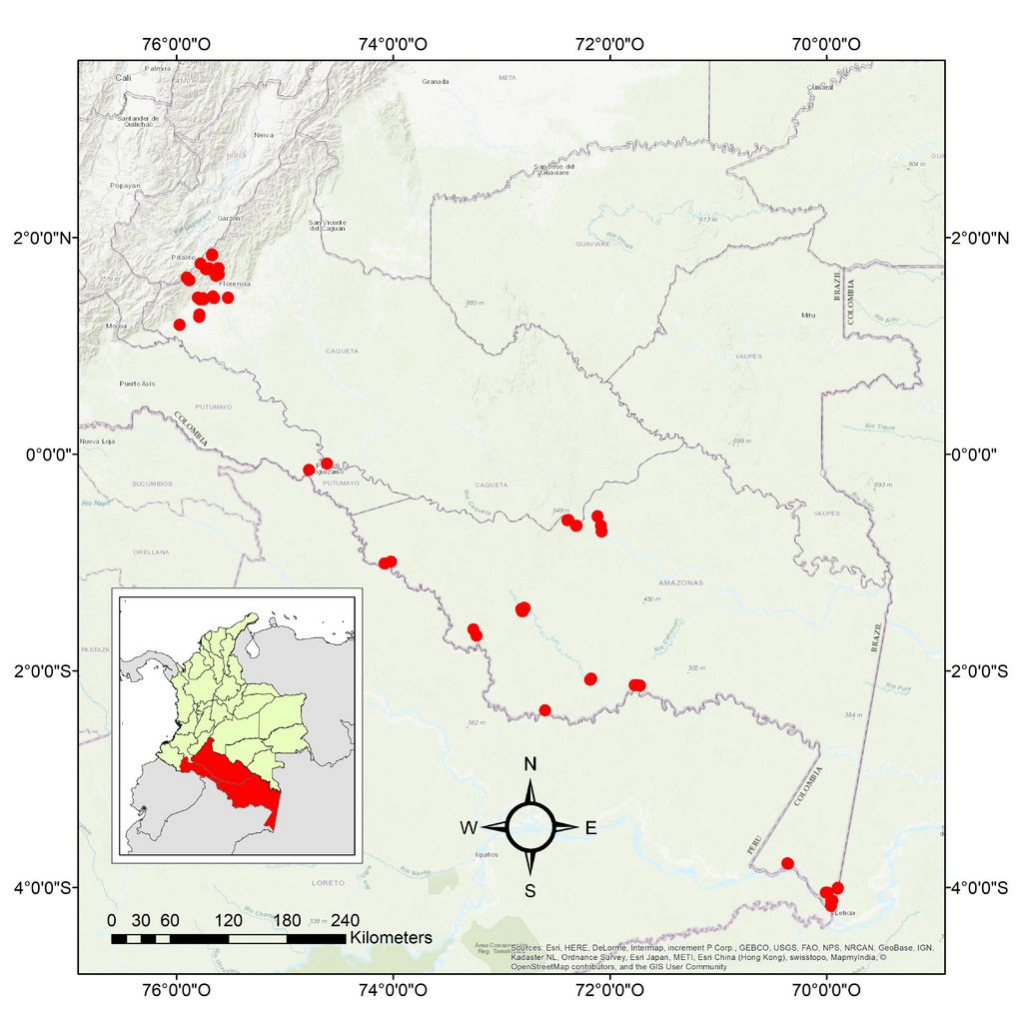
Study area, sampling localities.

Three Colombian states of the Amazon region were sampled: Amazonas, Caquetá and Putumayo (Fig. 1). The study area includes the Andean-Amazonian transition from north to south of the Colombian Amazon region up to the borders with Peru and Brazil along the Amazon River. Sampling altitude went from 78 to 2275 metres above sea level. There, 71 sampling sites in 13 municipalities were sampled: in Caquetá, the municipalities of Belén de los Andaquíes, Florencia, Morelia, San José and Solano; in Putumayo, the municipalities of Puerto Leguizamo and La Tagua; in Amazonas, the municipalities of El Encanto, La Chorrera, Leticia, Puerto Alegria, Puerto Arica, Puerto Nariño and Puerto Santander. Different natural and anthropic land uses were included in the sampling: primary and secondary forests, young secondary forests, pastures and indigenous slash-and-burn agricultural plots (Table 1).

### Sample collection and analysis

Soil ant collection took place between September 2015 and July 2017. Soil ants were collected using the methodology suggested by the Tropical Soil Biology and Fertility Program (TSBF) for soil macrofauna collection (Anderson and Ingram 1993). In each sampling site, a plot of 60 x 60 metres was selected. There, five monoliths of 25 x 25 x 30 cm of depth were done: one in each corner of the 60 x 60 m delimited square plot and one in the centre of it. In each monolith, macrofauna samples were collected at four depths: litter, 0 – 10 cm, 10 – 20 cm and 20 – 30 cm. Macrofauna collection in each monolith depth was undertaken in the field manually. Recovered samples were preserved in ethanol at 75% until their arrival to the SINCHI Institute laboratories in Leticia, Colombia, where specimens were vouchered and preserved in the CATAC collection.

In the laboratory, samples were cleaned and classified into morphotypes and species. All samples were identified by using the keys of recent revisions, verifying the species with the diagnosis and in some cases comparing with photos of type material in AntWeb (Brandão 1990, Kugler 1994, De Andrade and Baroni 1999, Palacio 1999, Fernández 2003, Longino and Fernández 2007, Jiménez et al. 2008, Mackay and Mackay 2010, Ortiz and Fernández 2011, Pacheco and Mackay 2013, Lenhart et al. 2013, Ješovnik and Schultz 2017, AntWeb 2018, LaPolla 2004, Snelling and Longino 1992, Brandão 2003, Longino 2010, Fernández et al. 2015, Lattke et al. 2007, Lattke 1997, Longino 2013, Sosa-Calvo et al. 2018, Longino 2003). *Camponotus, Brachymyrmex* and *Pheidole* were identified through the comparison of material identified by specialists and reference collection. All data were organised alphabetically by subfamily, genus and species in an ant checklist following the nomenclature suggested in the Bolton online catalogue of the ants of the world (AntCat, Bolton 2018).

## Analysis

### Checklist of the soil ant species of Colombian Amazon

A total of 1341 specimens and 4318 individuals were analysed. From the total soil macrofauna, ants were the most abundant and species-richest organisms collected. Ants dominated litter and 0 - 10 cm depths (Barros et al. 2002, Mathieu et al. 2005, Rossi et al. 2006, Velásquez et al. 2012, Suárez Salazar et al. 2015). Litter had the highest species richness with 129 species, followed by the 0 - 10 cm depth with 110 species. Layers from 10 - 20 cm depth and 20 - 30 cm depth had 77 and 45 species, respectively, showing a decreasing ant richness structure in the soil profile with depth.

The preliminary checklist of soil ants from the Colombian Amazon region (Table 2), contains 218 species distributed in 60 genera of 10 subfamilies. The richest subfamily was Myrmicinae with 99 species, followed by Ponerinae with 41 species. Other subfamilies found there included Formicinae with 31 species, Ectatomminae with 18 species, Dolichoderinae with 14 species, Pseudomyrmecinae with 6 species, Dorylinae with 5 species, Amblyopone with 2 species and the Paraponerinae and Proceratiinae with 1 species each, respectively. The richest genus was *Pheidole* Westwood, 1839 with 27 species, followed by *Crematogaster* Lund, 1831 with 16 species. Other genera rich in species are *Camponotus* Mayr, 1861 with 14 species, *Odontomachus* Latreille, 1804 with 10 species and *Gnamptogenys* Roger, 1863 with 8 species.

Ant richness in this report is remarkable when compared with previous reports from the Amazon region. The study done by Ryder Wilkie et al. 2010, which is recognised as the most complete work on ant diversity in the Amazon region, recorded at the Tiputini Reserve in Ecuador 66 genera and more than 300 species between subsoil and canopy. The high diversity reported in our work was certainly the effect of the broad area sampled (which includes three river basins) and the wide range of altitude included (Marsh et al. 2018).

The following are new records for Colombia:

*Acropyga tricuspis* (LaPolla, 2004)

**Specimen Data. 4 w**. AMAZONAS. Puerto Nariño [03°46'33.6"S; 70°21'41.8"W], 84 m a.s.l., 16 Jun 2017, C. Peña. Identification by D. Castro & A. Meneses (CATAC - 0413).

Comments. New record for Colombia. This species has been recorded in the Brazilian Amazonia (LaPolla 2004).

*Typhlomyrmex clavicornis* (Emery, 1906)

**Specimen Data. 3 w**. CAQUETÁ. Belén de los Andaquies [01°42'06.8"N; 75°53'57.5"W], 1500 m a.s.l., 23 Jan 2016, D. Castro. Identification by D. Castro & S. Sanchez (CATAC - 0893); 8 w, CAQUETÁ. Florencia, Palmichar [01°42'52.2"N; 75°36'53.6"W], 241 m a.s.l., 23 Mar 2016, Y. Virguez. Identification by D. Castro & S. Sanchez (CATAC - 0292).

Comments. New record for Colombia. This species has been recorded in Bolivia (Type locality), Brazil, French Guiana, Guyana, Paraguay and Suriname (Fernández and Arias-Penna 2008, Wild 2007).

*Typhlomyrmex meire* (Lacau, Villemant & Delabie, 2004)

**Specimen Data. 2 w.** CAQUETÁ: Florencia, Sebastopol [01°43'00.12"N; 75°36'49.3"W], 527 m a.s.l., 29 Mar 2016, Y. Virguez. Identification by D. Castro & S. Sanchez (CATAC-02563).

Comments. New record for Colombia. This species has been recorded in Brazil (Lacau et al. 2004).

*Cyphomyrmex bicornis* (Forel, 1895)

**Specimen Data. 1 w.** AMAZONAS. Leticia. Tanimboca Natural Reserve, [04°07'15.4"S - 69°57'19.7"W], 98 m a.s.l., 23 Jun 2017, D. Castro. Identification by M. Tocora (CATAC-01582).

Comments. New record for Colombia. This species has been recorded in Brazil (Type Locality) (Kempf 1966).

*Megalomyrmex emeryi* (Forel, 1904)

**Specimen Data. 6 w**. CAQUETÁ. Florencia, Sebastopol [01°43'00.12"N; 75°36'49.3"W], 527 m a.s.l., 29 Mar 2016, Y. Virguez. Identification by M. Tocora (CATAC-0326).

Comments. New record for Colombia. This species has been recorded in Bolivia, French Guiana, Guyana, Peru and Suriname (Type Locality) (Brandão 2003, Brandão 1990).

*Myrmicocrypta spinosa* (Weber, 1937)

**Specimen Data. 1 w.** CAQUETÁ. Florencia, Arandia [01°26'39.9"N - 75°31'29.1"Wˈ], 259 m a.s.l., 2 Jul 2016, Y. Virguez. Identification by M. Tocora (CATAC-0331).

Comments. New record for Colombia. This species has been recorded in Guyana (Type Locality) (Weber 1937).

## Discussion

The Caquetá river basin showed the highest number of soil ant species amongst basins (149 species, which corresponded to 68% of the total ants recorded), followed by the Amazon river basin (86 species, 40%) and the Putumayo river basin (71 species, 33%). From all species recorded, 89 species were exclusively registered in the Caquetá river basin, which was twice the number of species reported exclusively in the Amazon river basin (36 species) and in the Putumayo river basin (25 species). The high diversity of the Caquetá river basin may be areflection of the geographic conditions of the area and the sampling effort used there. The Caquetá river basin includes the Andean-Amazonian transition where a high turnover of species might occur, but additionally, it was the one with the greatest sampling effort.

From all the ant species recorded, 20 species were found in the four soil depths (Table 2). The most abundant of these species were: *Wasmannia auropunctata* Roger, 1863, *Tranopelta gilva* Mayr, 1866, *Sericomyrmex bondari* Borgmeier, 1937, *Crematogaster limata* Smith, 1858, *Crematogaster carinata* Mayr, 1862, *Crematogaster brasiliensis* Mayr, 1878, *Crematogaster abstinens* Forel, 1899 and *Brachymyrmex cordemoyi* Forel, 1895.

The genera *Acropyga* and *Typhlomyrmex* are underground genera commonly collected at deep soil depths. For example, the genus *Typhlomyrmex* was collected mostly at 10 - 20 and 20 – 30 cm soil depths. Although TSBF was appropriate for collecting these soil ants, which are generally undersampled with other methods of collection, the TSBF method might underestimate army ants and other large ants such as Paraponerinae that were not recorded in the searched Amazon basin area (Ryder Wilkie et al. 2007, Oliveira and Morato 2009, Sanabria-Blandón and de Ulloa 2011, Ryder Wilkie et al. 2010). However, the use of soil monoliths for macrofauna sampling allowed comparisons between macrofauna groups (e.g. ants with other macrofauna groups). The use of more than one method to obtain an accurate image of the community of ants has been proven (e.g. Winkler or *pitfall* for estimating the abundance of leaf litter ants) (Delsinne and Arias-Penna 2012, Wong and Guénard 2017, Ryder Wilkie et al. 2007). However, in this study, the composition of subterranean ant subfamilies was not affected by the method of collection used (TSBF) as the proportions of subfamilies were similar to those obtained using additional methods (Wong and Guénard 2017).

An important number of canopy and arboreal ant species such as *Crematogaster, Azteca, Dolichoderus, Camponotus* and *Cephalotes* were collected. Studies of ant fauna in the Colombian Amazon (Pérez et al. 2009) highlighted the diversity of these genera in the region. Canopy and arboreal ant species can be an important part of the ant density in the upper strata of soil (leaf litter and the depth of 0 - 10 cm) as occurred in this study where most of these ants were collected in litter. Results inferred that these ants use the soil as a way for transportation or for food provisioning, although they do not live in the soil such as ants of the genera *Pheidole, Acropyga, Cryptopone* or *Hypoponera*.

Some arboreal species of *Crematogaster, Camponotus, Myrmelachista, Procryptocerus* and *Pseudomyrmex* were found in soil deep horizons, even at 20-30 cm depth such as *Azteca* and *Pseudomyrmex.* Ant collection was done manually in the field. During this process, some arboreal ants could fall down and contaminate monolith samples when the bags were not well closed. However, arboreal ants may realistically be away from their common substrate or nest, as little is known about their biology, even more so when this is not the first time they have been recorded in soil samples (Rosumek et al. 2008, Vasconcelos et al. 2003, Delabie and Fowler 1995).

Ants are the most diverse soil macrofauna group in the Amazon region ([Bibr B4525218][Bibr B4684341]) and represent a high density (Table 3). In the Caquetá basin, they are the most dense organisms of the soil macrofauna. In the other two river basins, ants are only exceeded by termites. Differences in ant and termite densities might be a reflection of the land use sampled. Termites tend to be more abundant in less disturbed ecosystems (Mboukou-Kimbatsa et al. 1998, Velásquez et al. 2012), while ants tend to be more abundant in disturbed or degraded ecosystems of the Amazon region. In our study, the Caquetá basin is where the most disturbed coverings, such as pastures and young secondary forests, are found (Table 1) (Aquino et al. 2008, Barros et al. 2002, Marichal et al. 2014, Pinzón et al. 2014, Rousseau et al. 2014).

The Neotropics (including the Amazon basin) have been recognised as a region of speciation and conservation of multiple lineages of ants (Moreau and Bell 2013). Results presented here increase the knowledge of soil ants from the Amazon region and suggest that ant species richness may increase considerably when sampling effort increases and combined methodologies are used to capture ants in different habitats.

## Figures and Tables

**Table 1.** List of TSBF monoliths sampling sites in Colombian Amazon soils.

**River basin**	**State**	**Town**	**Land use**	**Altitude**	**Latitude**	**Longitude**
Amazonas	Amazonas	Leticia	Primary forest	80	S4°10'09", W69°57'25"	
			Primary forest	81	S4°10'09.1", W69°57'27.2"	
			Primary forest	98	S04°07'15.4", W69°57'19.7"	
			Primary forest	106	S04°02'45.7", W69°59'26.8"	
			Primary forest	110	S04°00'32.5", W69°53'43.3"	
			Primary forest	119	S04°00'10.5", W69°53'47.6"	
			Primary forest	121	S04°02'48.0", W70°00'20.4"	
			Secondary forest	87	S04°07'14.7", W69°56'40.9"	
		Pto. Nariño	Secondary forest	84	S03°46'33.6", W70°21'41.8"	
			Young secondary forest	102	S03°46'52.6", W70°21'17.7"	
Caquetá		Pto. Santander	Secondary forest	116	S00°39'43.3", W72°18'38.2"	
	Caquetá	Belen	Pasture	233	N01°16ˈ08.3", W75°47ˈ17.6"	
			Pasture	242	N01°15ˈ59.9", W75°47ˈ23.4"	
			Primary forest	500	N01°36'17.8", W75°52'50.9"	
			Primary forest	625	N01°36'28.6", W75°53'12.6"	
			Primary forest	750	N01°37'50.3", W75°54'21.3"	
			Primary forest	875	N01°37'40.0", W75°54'16.8"	
			Primary forest	1000	N01°37'27.4", W75°54'04.3"	
			Primary forest	1125	N01°40'14.4", W75°54'13.3"	
			Primary forest	1247	N01°50'36.4", W75°40'18.3"	
			Primary forest	1250	N01°40'45.2", W75°54'12.4"	
			Primary forest	1375	N01°40'54.3", W75°54'17.1"	
			Primary forest	1500	N01°42'06.8", W75°53'57.5"	
			Primary forest	1625	N01°41'49.9", W75°54'18.1"	
			Primary forest	1875	N01°43'04.4", W75°54'11.7"	
			Young secondary forest	250	N01°25'46.2", W75°45'01.01"	
			Young secondary forest	251	N01°25'57,6", W75°45'06,4"	
			Young secondary forest	268	N01°26'45.7", W75°48'12.4"	
			Young secondary forest	271	N01°25'42,7", W75°46'56,3"	
		Florencia	Pasture	383	N01°38ˈ54,1", W75°38ˈ13,6"	
			Pasture	437	N01°39'15.6", W75°38'00.2"	
			Pasture	527	N01°43'00.12", W75°36'49,3"	
			Pasture	2275	N01°45'33.7", W75°46'41.5"	
			Pasture	981	N01°42'29.8", W75°41'32.4"	
			Pasture	1268	N01°42'55.1", W75°42'06.0"	
			Secondary forest	259	N01°26ˈ39.9", W75°31ˈ29.1"	
			Secondary forest	488	N01°40'35.0", W75°37'5.86"	
			Secondary forest	495	N01°42'26.8", W75°36'59.5"	
			Secondary forest	598	N01°43'04.0", W75°36'45.6"	
			Secondary forest	1328	N01°42'37.7", W75°43'49.1"	
			Secondary forest	1571	N01°50ˈ09,0", W75°40ˈ19,2"	
			Young secondary forest	241	N01°42'52.2", W75°36'53.6"	
			Young secondary forest	246	N01°42'27.6", W75°43'26.0"	
			Young secondary forest	260	N01°26ˈ40.9", W75°31ˈ32.1"	
			Young secondary forest	425	N01°40'47.0", W75°37'48.3"	
			Young secondary forest	506	N01°42'27.9", W75°36'59.7"	
			Young secondary forest	1617	N01°50'36.9", W75°40'16.1"	
		Morelia	Pasture	252	N01°27'21.63", W75°39'48.10"	
			Secondary forest	249	N01°26ˈ28.8", W75°39ˈ10.3"	
			Young secondary forest	261	N01°26'18.1'", W75°45'16.3"	
			Young secondary forest	555	N01°26ˈ29.9", W75°39ˈ12.5"	
			Young secondary forest	248	N01°39'35.2", W75°36'33.9"	
		San José	Primary forest	284	N01°11ˈ38.4", W75°58ˈ16.7"	
			Young secondary forest	288	N01°11ˈ40.1", W75°58ˈ18.7"	
		Solano	Young secondary forest	106	S00°34'30.8", W72°06'51"	
Putumayo	Amazonas	El Encanto	Primary forest	140	S01°37'03.7", W73°15'31.7"	
			Primary forest	141	S01°40'34.7", W73°13'51.4"	
		La Chorrera	Primary forest	126	S02°04'55.2", W72°10'54.8"	
			Primary forest	133	S02°04'14.4", W72°10'14.2"	
			Primary forest	146	S01°26'54.2", W72°48'13.3"	
			Primary forest	151	S01°26'56.3", W72°48'37"	
			Primary forest	154	S01°25'05.7, W72°47'21.2"	
			Secondary forest	147	S01°25'11", W72°47'10.5"	
		Pto. Alegria	Primary forest	154	S01°00'31.5", W74°04'44.5"	
			Primary forest	169	S00°59'34.3", W74°01'10.4"	
		Pto. Arica	Primary forest	108	S02°07'55.6", W71°44'42.8"	
			Primary forest	120	S02°07'59", W71°46'54"	
			Primary forest	127	S02°08'10.5", W71°43'16.8"	
		Sabalo	Primary forest	142	S02°21'11.7", W72°35'53.4"	
	Putumayo	Pto. Leguizamo	Secondary forest	182	S00°05'14.9", W74°36'38.4"	
			Secondary forest	213	S00°08'42.1", W74°46'40.9"	

**Table 2.** Checklist of the soil ant species of the Colombian Amazon. The list is organised alphabetically by subfamily, genus and species. Species names in bold characters refer to species recorded for the first time in Colombia. River basins corresponded to: A = Amazon river; C = Caquetá river; P = Putumayo river. Depth of species collection: 1 = Litter; 2 = 0 – 10 cm; 3 = 10 – 20 cm; 4 = 20 – 30 cm. Land use corresponded to PF = Primary forest; SF = Secondary forest; P = Pasture; R = Young secondary regeneration forest.

**Subfamilies**	**Scientific valid name**	**River basin**	**Depth**	**Land use**
Amblyoponinae	*Prionopelta antillana* Forel, 1909	A,C	1,2,3	R
	*Fulakora orizabana* (Brown, 1960)	C	3	P
Dolichoderinae	*Azteca* sp1	C,P	1,2	PF
	*Azteca* sp2	A,P	1,2,4	PF, R
	*Azteca* sp3	A,C,P	1,2,3,4	PF, P, R
	*Azteca* sp4	C	2,3	SF
	*Azteca* sp5	C	1,4	PF, P
	*Dolichoderus attelaboides* Fabricius, 1775	A	1	PF
	*Dolichoderus bidens* Linnaeus, 1758	C,P	1,2	P, R
	*Dolichoderus bispinosus* Olivier, 1792	P	1	PF, R
	*Dolichoderus imitator* Emery, 1894	A,C	2,3	R
	*Dolichoderus quadridenticulatus* Roger, 1862	C	2	P
	*Dolichoderus rugosus* Smith, 1858	A,P	1	PF, R
	*Linepithema* sp1	A,C,P	1,2,3,4	PF, P
	*Linepithema* sp2	C	1,2,3,4	PF, P, SF
	*Linepithema* sp3	C	1,2,3,4	PF, SF
Dorylinae	*Cheliomyrmex andicola* Emery, 1894	C	2	SF
	*Eciton hamatum* Fabricius, 1782	A,C	1	PF
	*Labidus praedator* Smith, 1858	C	1,2,3	P, SF
	*Leptanilloides* sp.	P	2	PF
	Neivamyrmex cf. hetschkoi Mayr, 1886	C	1,4	PF, SF
Ectatomminae	*Ectatomma brunneum* Smith, 1858	A,C	1	PF, R
	*Ectatomma edentatum* Roger, 1863	A	2	PF
	*Ectatomma lugens* Emery, 1894	P	2	PF
	*Ectatomma ruidum* Roger, 1860	A,C	1,2,3	PF, P
	*Ectatomma tuberculatum* Olivier, 1792	C	2	PF
	Gnamptogenys cf. ilimani Lattke, 1995	C	1	P
	*Gnamptogenys* (gr. minuta) sp	A	2	PF
	*Gnamptogenys kempfi* Lenko, 1964	A	2	PF
	Gnamptogenys cf. lavra Lattke, 2002	A,C,P	1,2	PF
	*Gnamptogenys porcata* Emery, 1896	C	3	R
	*Gnamptogenys striatula* Mayr, 1884	C,P	1,3	PF
	*Gnamptogenys strigata* Norton, 1868	P	3	PF
	*Gnamptogenys tortuolosa* Smith, 1858	A	1	PF
	*Typhlomyrmex clavicornis* Emery, 1906	C,P	3,4	PF, SF
	*Typhlomyrmex major* Santschi, 1923	A,C	3,4	PF, SF
	*Typhlomyrmex meire* Lacau, Villemant & Delabie, 2004	C	1,3	P
	*Typhlomyrmex pusillus* Emery, 1894	C	1,2,4	PF, P
	*Typhlomyrmex* sp.	A,C	2	PF
Formicinae	Acropyga aff. epedana Snelling, 1973	C	2	R
	*Acropyga exsanguis* Wheeler, 1909	C	4	PF, R
	*Acropyga goeldii* Forel, 1893	C,P	1,2,3	PF
	*Acropyga guianensis* Weber, 1944	P	1,2,3	PF, P, SF
	*Acropyga smithii* Forel, 1893	P	2	PF
	*Acropyga tricuspis* LaPolla, 2004	A	1,2	R
	Brachymyrmex aff. heeri Forel, 1874	P	2	PF
	Brachymyrmex aff. australis Forel, 1901	C	1	P
	*Brachymyrmex cordemoyi* Forel, 1895	A,C	1,2,3,4	PF, SF
	*Brachymyrmex myops* Emery, 1906	A	2	PF
	*Brachymyrmex pictus* Mayr, 1887	C	1	SF
	Camponotus aff. ager Smith, 1858	A	2	PF
	*Camponotus atriceps* Smith, 1858	A	1	PF
	*Camponotus casicus* Santschi, 1920	C	1	SF
	*Camponotus femoratus* Fabricius, 1804	A,C,P	1,2	PF, P, SF
	*Camponotus latangulus* Roger, 1863	C	1	P
	*Camponotus nitidior* Santschi, 1921	C	2	PF
	*Camponotus novogranadensis* Mayr, 1870	A	1	PF
	*Camponotus rapax* Fabricius, 1804	C	1,3	PF
	*Camponotus rufipes* Fabricius, 1775	C	1	PF
	*Camponotus senex* Smith, 1858	C	3	P
	*Camponotus* sp1	C	1	SF
	*Camponotus* sp2	A	1	PF
	*Camponotus* sp3	C	3	R
	*Camponotus* sp4	C	2	PF
	*Gigantiops destructor* Fabricius, 1804	A,C,P	1,2	PF
	*Myrmelachista* sp.	C	1,3	PF
	*Nylanderia* sp1	A	1,2,3,4	PF, P, R, SF
	*Nylanderia* sp2	A,C	1,2,3,4	PF, P, S, SF
	*Nylanderia* sp3	A	1,2	PF, P, SF
	*Nylanderia* sp4	A	3	PF
Myrmicinae	*Acromyrmex coronatus* Fabricius, 1804	C	4	PF
	*Apterostigma auriculatum* Wheeler, 1925	P	2	PF
	Apterostigma cf. acre Lattke, 1997	A	1	R
	*Apterostigma goniodes* Lattke, 1997	C	1,4	PF
	*Apterostigma* (gr. pilosum) sp.1	A	1	SF
	*Apterostigma* (gr. pilosum) sp.2	C	2	R
	*Apterostigma megacephala* Lattke, 1999	C	1	P
	*Atta colombica* Guérin-Méneville, 1844	C	1	PF, P
	*Blepharidatta brasiliensis* Wheeler, 1915	A	1	PF
	*Cardiocondyla nuda* Mayr, 1866	C	1	SF
	*Carebara brevipilosa* Fernández, 2004	C	3	P
	*Carebara* (gr. escherichi) sp.1	A	4	PF
	Cephalotes aff. cordatus Smith, 1853	C	2	P
	*Cephalotes atratus* Linnaeus, 1758	A,C	1	PF
	Cephalotes cf. patellaris Mayr, 1866	C	1	SF
	*Cephalotes spinosus* Mayr, 1862	C	1,3	P
	*Crematogaster abstinens* Forel, 1899	A,C	1,2,3,4	SF
	*Crematogaster acuta* Fabricius, 1804	A	2,3,4	PF
	Crematogaster aff. evallans Forel, 1907	C	2,3	SF
	*Crematogaster brasiliensis* Mayr, 1878	A,C	1,2,3,4	PF, S, SF
	*Crematogaster bryophilia* Longino, 2003	A	1	PF
	*Crematogaster carinata* Mayr, 1862	A,C,P	1,2,3,4	PF, P, SF
	Crematogaster cf. snellingi Longino, 2003	A	1	PF
	*Crematogaster crinosa* Mayr, 1862	C	1,3,4	SF
	*Crematogaster erecta* Mayr, 1866	C	2	SF
	*Crematogaster flavosensitiva* Longino, 2003	P	1	PF
	*Crematogaster limata* Smith, 1858	A,C,P	1,2,3,4	PF, P, R, SF
	*Crematogaster longispina* Emery, 1890	A,C	1,2	PF, SF
	*Crematogaster minutissima* Mayr, 1870	A	1,2	PF
	*Crematogaster nigropilosa* Mayr, 1870	A	2,3	PF
	*Crematogaster sotobosque* Longino, 2003	C,P	2,3	PF
	*Crematogaster tenuicula* Forel, 1904	A,P	1,2,3	PF, R
	*Cyphomyrmex bicornis* Forel, 1895	A	2	PF
	*Cyphomyrmex laevigatus* Weber, 1938	A,P	1	PF, R
	*Cyphomyrmex minutus* Mayr, 1862	C	1	PF
	*Cyphomyrmex peltatus* Kempf, 1966	C	1	PF
	*Cyphomyrmex rimosus* Spinola, 1851	C,P	1,2,3	PF, P, SF
	*Hylomyrma immanis* Kempf, 1973	A,C	1,2	PF, SF
	*Hylomyrma sagax* Kempf, 1973	C	1	PF
	*Kempfidris inusualis* Fernández, 2007	A	2	R
	Megalomyrmex cf. balzani Emery, 1894	C	3	PF
	*Megalomyrmex emeryi* Forel, 1904	C,P	1,2,3	P
	*Megalomyrmex foreli* Emery, 1890	C,P	1,2,3	PF, P
	*Megalomyrmex leoninus* Forel, 1885	C	1,3	P
	*Megalomyrmex megadrifti* Boudinot, Sumnicht & Adams, 2013	C	1	PF
	*Mycocepurus smithii* Forel, 1893	A,C,P	1,2,3	PF, SF
	*Myrmicocrypta longinoda* Weber, 1938	A	2	PF
	*Myrmicocrypta* sp.	C	2	PF
	*Myrmicocrypta spinosa* Weber, 1937	C	3	PF
	*Nesomyrmex tristani* Emery, 1896	C	1	SF
	*Ochetomyrmex neopolitus* Fernández, 2003	P	1	PF
	*Octostruma balzani* Emery, 1894	C	1	PF
	*Octostruma impressa* Palacio, 1997	C	1	PF
	Pheidole aff. biconstricta Mayr, 1870	A,C,P	1,2	PF
	Pheidole aff. chocoensis Wilson, 2003	P	1,3	PF
	Pheidole aff. cocciphaga Borgmeier, 1934	A	1	PF
	Pheidole aff. huilana Wilson, 2003	P	1,2	PF, R
	Pheidole aff. radoszkowski Mayr, 1884	C	3	SF
	Pheidole aff. sensitiva Borgmeier, 1959	P	1	PF
	Pheidole aff. subnuda Wilson, 2003	P	1	PF
	Pheidole aff. vafra Santschi, 1923	C	2	SF
	*Pheidole astur* Wilson, 2003	P	3	PF
	*Pheidole gertrudae* Forel, 1886	C	3	PF
	*Pheidole* sp1	C	1	SF
	*Pheidole* sp2	A,C	1,2,3	PF, SF
	*Pheidole* sp3	P	2	PF
	*Pheidole* sp4	P	4	PF
	*Pheidole* sp5	C	3	P, SF
	*Pheidole* sp6	C	1	SF
	*Pheidole* sp7	C	2	SF
	*Pheidole* sp8	P	2,3	PF
	*Pheidole* sp9	C	3,4	P, SF
	*Pheidole* sp10	C	2	P
	*Pheidole* sp11	C	2	SF
	*Pheidole* sp12	C	1	SF
	*Pheidole* sp13	C	2	P
	*Pheidole* sp14	A,P	2,3,4	PF, SF
	*Pheidole* sp15	A,P	1,3	PF
	*Pheidole* sp16	A	1,2,4	PF
	*Pheidole* sp17	A	4	R
	*Procryptocerus scabriusculus* Forel, 1899	C	3	PF
	*Rogeria belti* Mann, 1922	A	1,2	PF, P
	*Sericomyrmex bondari* Borgmeier, 1937	A,C	1,2,3,4	PF, R
	*Solenopsis geminata* Fabricius, 1804	C	1,2,3	PF, P, R
	*Solenopsis* sp1	C	1	P
	*Solenopsis* sp2	P	2	PF
	*Solenopsis* sp3	A,P	1,2,4	PF, P, SF
	*Solenopsis* sp4	A,C	1,3	PF, P, SF
	*Solenopsis* sp5	C	1,2,3,4	PF, P, SF
	*Solenopsis virulens* Smith, 1858	P	1	PF
	*Strumigenys denticulata* Mayr, 1887	A	1	R
	*Strumigenys interfectiva* Lattke & Goitía, 1997	C	1	PF
	*Strumigenys smithii* Forel, 1886	P	1	PF
	*Trachymyrmex* sp1	C,P	1,2,3,4	PF, SF
	*Trachymyrmex* sp2	A,C,P	1,2,3,4	PF, P, SF
	*Trachymyrmex* sp3	C,P	1,3,4	PF, SF
	*Trachymyrmex* sp4	C	3	PF
	*Tranopelta gilva* Mayr, 1866	A,C,P	1,2,3,4	PF, P, SF
	*Wasmannia auropunctata* Roger, 1863	A,C,P	1,2,3,4	PF, P, R, SF
Paraponerinae	*Paraponera clavata* Fabricius, 1775	C,P	1	PF
Ponerinae	*Anochetus diegensis* Forel, 1912	C	1	PF, P
	*Anochetus mayri* Emery, 1884	C	1	PF
	Anochetus cf. neglectus Emery, 1894	C	1	PF
	*Centromyrmex alfaroi* Emery, 1890	C	2	SF
	*Centromyrmex brachycola* Roger, 1861	A,C,P	1,2,3	PF, P, SF
	*Cryptopone guianensis* Weber, 1939	C,P	2,3	PF
	*Cryptopone holmgreni* Wheeler, 1925	C	2,3,4	PF, SF
	*Dinponera longipes* Emery, 1901	A	1	PF
	*Hypoponera distinguenda* Emery, 1890	A,C	1,2	PF, R
	*Hypoponera* sp1	C,P	1,2,3,4	PF, P, R
	*Hypoponera* sp2	C,P	1,2,3	PF, R
	*Hypoponera* sp3	A,P	1,2	PF, R
	*Hypoponera* sp4	A,C,P	1,2,3	PF, SF
	*Hypoponera* sp5	A,C,P	1,2,3	PF, P, SF
	*Hypoponera* sp6	A,C	1,2,3,4	PF, P, R, SF
	*Leptogenys* (gr. crudelis) sp	C	1	PF
	*Mayaponera constricta* Mayr, 1884	A,C,P	1,2	PF, P, R, SF
	*Neoponera apicalis* Latreille, 1802	A,C	1	PF
	*Neoponera commutata* Roger, 1860	P	2	PF
	*Neoponera unidentata* Mayr, 1862	C	3	PF
	*Neoponera verenae* Forel, 1922	C	1	PF
	*Neoponera villosa* Fabricius, 1804	A	1	PF
	Odontomachus aff. panamensis Forel, 1899	C	1	SF
	*Odontomachus bauri* Emery, 1892	C	1,4	PF, P
	*Odontomachus bradleyi* Brown, 1976	C	1	PF
	*Odontomachus caelatus* Brown, 1976	P	1	PF
	*Odontomachus haematodus* Linnaeus, 1758	A,C	1	SF
	*Odontomachus meinerti* Forel, 1905	C	1,3	PF
	*Odontomachus opaciventris* Forel, 1899	C,P	1	PF, SF
	*Odontomachus scalptus* Brown, 1978	C	1	SF
	*Odontomachus spisuss* Kempf, 1962	P	1	PF
	Odontomachus cf. yucatecus Brown, 1976	C	2	PF
	*Pachycondyla crassinoda* Latreille, 1802	P	1,2	PF
	*Pachycondyla fuscoatra* Roger, 1861	A	1	R
	*Pachycondyla harpax* Fabricius, 1804	A,C,P	1,2	PF, R
	*Pachycondyla impressa* Roger, 1861	A	1	PF
	*Pseudoponera stigma* Fabricius, 1804	A,C,P	1,2	PF
	*Rasopone arhuaca* Forel, 1901	A,C,P	1,2,3	PF, R
	*Rasopone becculata* MacKay & MacKay, 2010	C	2,3,4	PF
	*Rasopone lunaris* Emery, 1896	A	2	PF
	*Rasopone* sp.	C	2	PF
Proceratiinae	*Proceratium transitionis* de Andrade, 2003	C	1	PF
Pseudomyrmecinae	*Pseudomyrmex* sp1	A,C	1,2,3	PF, P
	*Pseudomyrmex* sp2	A,C,P	1,2,4	PF, P, R, SF
	*Pseudomyrmex* sp3	C	1,2,3,4	PF, P, SF
	*Pseudomyrmex* sp4	C,P	1,2,4	PF, SF
	*Pseudomyrmex* sp5	C	1,3,4	P, SF
	*Pseudomyrmex* sp6	C,P	1	PF

**Table 3.** Density (Individuals/m^2^) of the main taxonomic groups collected in TSBF monoliths in the Colombian Amazon.

**Taxa Group**	**River Basin**		
	**Amazonas**	**Caquetá**	**Putumayo**
Formicidae	274.16	173.70	82.12
Amblyoponinae	0.78	0.49	0.00
Dolichoderinae	4.39	9.66	3.29
Dorylinae	0.00	2.47	0.47
Ectatomminae	7.24	4.23	3.29
Formicinae	72.87	37.94	9.18
Myrmicinae	145.74	91.96	45.88
Paraponerinae	0.00	0.63	0.71
Ponerinae	41.34	24.26	18.82
Proceratiinae	0.00	0.07	0.00
Pseudomyrmecinae	1.29	1.97	0.47
Termitoidea	289.15	146.33	142.12
Coleoptera	33.59	15.80	34.12
Araneae	26.36	15.87	17.18
Immature insects	18.35	15.02	13.18
Chilopoda	15.50	8.32	12.00
Diplopoda	11.11	6.98	9.41
Blattodea	4.13	7.62	4.94
Hemiptera	9.30	5.71	5.18
Isopoda	8.79	3.74	8.94
Diplura	8.01	3.95	4.00
Opiliones	8.01	3.10	4.24
